# Self-Replication of Localized Vegetation Patches in Scarce Environments

**DOI:** 10.1038/srep33703

**Published:** 2016-09-21

**Authors:** Ignacio Bordeu, Marcel G. Clerc, Piere Couteron, René Lefever, Mustapha Tlidi

**Affiliations:** 1 Departamento de Física, Facultad de Ciencias, Universidad de Chile, Casilla 653, Santiago, Chile; 2 Departamento de Física, Facultad de Ciencias Físicas y Matemáticas, Universidad de Chile, Casilla 487-3, Santiago, Chile; 3 IRD, UMR AMAP, c/o Cirad, 34000 Montpellier, France; 4 Faculté des Sciences, Université Libre de Bruxelles (ULB), Campus Plaine, CP. 231, B-1050 Bruxelles, Belgium

## Abstract

Desertification due to climate change and increasing drought periods is a worldwide problem for both ecology and economy. Our ability to understand how vegetation manages to survive and propagate through arid and semiarid ecosystems may be useful in the development of future strategies to prevent desertification, preserve flora—and fauna within—or even make use of scarce resources soils. In this paper, we study a robust phenomena observed in semi-arid ecosystems, by which localized vegetation patches split in a process called self-replication. Localized patches of vegetation are visible in nature at various spatial scales. Even though they have been described in literature, their growth mechanisms remain largely unexplored. Here, we develop an innovative statistical analysis based on real field observations to show that patches may exhibit deformation and splitting. This growth mechanism is opposite to the desertification since it allows to repopulate territories devoid of vegetation. We investigate these aspects by characterizing quantitatively, with a simple mathematical model, a new class of instabilities that lead to the self-replication phenomenon observed.

In arid and semi-arid landscapes around the world, it is common to encounter non-uniform vegetation covers exhibiting large spatial structures, generically called vegetation patterns^1^,^2^,^3^. These landscapes are characterised by either water limited resources and/or nutrient-poor territories. In the former case, the potential evapo-transpiration of the plants exceeds the water supply provided by rainfalls. At the level of individual plant, the water scarcity provokes an hydric stress that affects both the plants survivability and growth rate. At the community level, this hydric stress promotes clustering behaviour which induces spatial landscapes fragmentation. It is now generally admitted that this adaptation to hydric stress involves a symmetry-breaking modulational instability leading to the establishment of a stable periodic spatial patterns^4^,^5^,^6^,^7^,^8^,^9^,^10^,^11^,^12^,^13^,^14^,^15^,^16^,^17^,^18^,^19^,^20^,^21^,^22^,^23^,^24^,^25^,^26^.

Vegetation patterns are not always periodic. The spatial distribution of vegetation may consists of isolated or randomly distributed patches or gaps. Such irregular patterns can involve groves within grasslands^27^,^28^,^29^,^30^ or spots of bare soil within a grass matrix^31^. They consist of patches which are either isolated or forming clusters. In both cases, such patterns have been interpreted as localized structures^9^,^27^,^28^,^29^,^30^,^31^.

The aperiodic patterning phenomenon is not specific to peculiar soils or plant species. Localized vegetation patches or gaps may develop on soil ranging from sandy and silty to clayey, the nature of vegetation may consist of grasses, shrubs and trees. The extension of a patch can vary from small clumps of grasses (0.5–2 m^2^) to large groves of mulga (Acacia aneura) trees (100–1000 m^2^), such as those observed in central Australia^32^. On the other hand, the formation of localized patterns is an important issue not only in plant ecology context and environmental sciences but also it is a multidisciplinary area of research involving physics, chemistry and mathematics^33^.

Localized vegetation patches may exhibit a curvature instability that leads to a splitting of the patch into two new patches. Examples of such behaviour are shown in Fig. 1 and can also be observed in structures of tenths of meters in diameter in Zambia, Southern Africa (−13.787178°, 25.283842°). This intriguing phenomenon often called spot-replication or fingering is well documented in the context of magnetic fluids^34^, liquid crystals^35^,^36^, chemical systems^37^,^38^,^39^,^40^,^41^,^42^,^43^,^44^,^45^,^46^,^47^,^48^, in plant ecology^49^, material science^50^,^51^, granular fluid systems^52^,^53^, and nonlinear optics^54^. The fingering instability of planar fronts leading to the formation of labyrinth structures has been reported^55^. Similar phenomenon has been observed in fingering instability of localized structures^56^.

In this article, we investigate the self-replication mechanism in the context of natural vegetation ecosystems. We show that this phenomena is robust as it is observes in a wide range of species and size scales. By analysing satellite images from the semiarid ecosystem of the Catamarca region, Argentina, we show the emergence of characteristic statistical distributions of the vegetation patches and their spatial organisation. We consider a general interaction-redistribution model, where analytical and numerical results show that there exist a critical value of the level of the aridity under which a single circular vegetation patch destabilises, the curvature instability leads to an elliptical deformation followed by patch multiplication. This process continue in time until the system reaches a self-organized vegetation pattern in an hexagonal form. To compare field and numerical observations, we construct an initial condition for the simulations given by randomly distributed patches (which mimic long-range seed spreading), each patch is considered to be in a different stage of the self-replication process (this mimics the different ages of each patch). Under these considerations, we obtain a fair agreement between field and numerical observations, showing how self-replication is one of the mechanisms that mediate the spatial distribution and propagation of the vegetation in scarce environments.

This work is organized as follow: first, we study the spatial distribution and self-replication process in a real ecosystem by remote sensing imagery; secondly, we present a simple mathematical model, which is suitable for the description of vegetation pattern formation in semi-arid ecosystem and exhibits self-replication; thirdly, by numerical simulations and comparison to field observations, we show that self-replication can be an important mechanism for the phytomass repopulation; finally, we present the conclusions and projections of our work.

## Field observations of self-replication

Andes highlands are semi-arid ecosystems with a low amount of available resources. In particular, the Catamarca region in NW-Argentina (−23.436253°, −65.976767° at 3424 m a.s.l.), presents an average annual rainfall that reaches 369 mm (source: grid of climate observations CRU CL 2.0^57^), with a maximum in January of 71 mm and a minimum in July of 6 mm, temperatures vary from warm in the day to sub-zero in the night. Here, it is well known that *Festuca orthophylla* which produces tall, evergreen tussocks dominates the landscape over extended areas and periods of time at elevations between 3225 m and 4860 m a.s.l^58^,^59^,^60^. This specie is present in a variety of cold climates, adapting to diverse rainfall and soil moisture conditions. Festuca tussock arrange in circular shape compact structures composed by thousands of tightly packed tillers. The size of the tussocks depends on the resources available and weather conditions of their location, for instance, in western Bolivia they can reach 1.6 m high^59^,^60^. An important characteristic of Festuca is their shallow rooting system, which has been reported to cover an area 6-fold the area of the above ground canopy^59^,^60^. This quality allows each plant to have access to the resources in a total area equivalent to 6-fold the area of the projected canopy. This root network is the most important mechanism to capture resources in scarce environments, and also allows tussock-tussock competition for resources. This competitive interactions will be important throughout this article in understanding the observed spatial organisation of the tussocks.

This site, was selected in order to have a minimal slope, and no topographic perturbations such as mountains, canyons, rivers or highways. The region studied here covers an area of 109.4 km^2^ (384 m × 285 m), the image corresponding to the site was obtained using Google Earth Pro and consisted on a 4800 × 3562 pixels image.

**Figure 1:**
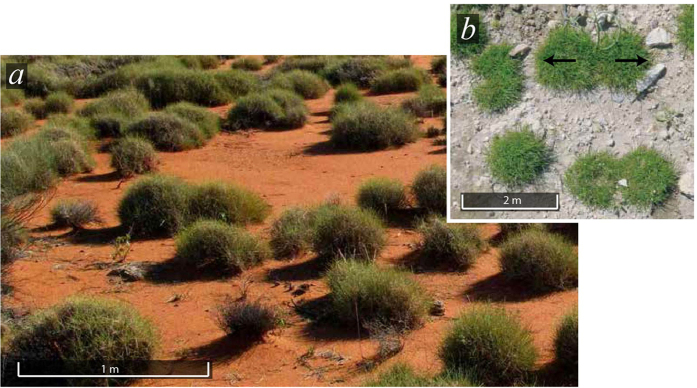
Localized patch instability. (**a**) Spinifex grassland, Yakabindi station, Western Australia (courtesy of Vilis Nams, Dalhousie University, Canada). (**b**) Patterns of P. bulbosa observed in the Northern Negev (reprinted from^49^).

**Figure 2:**
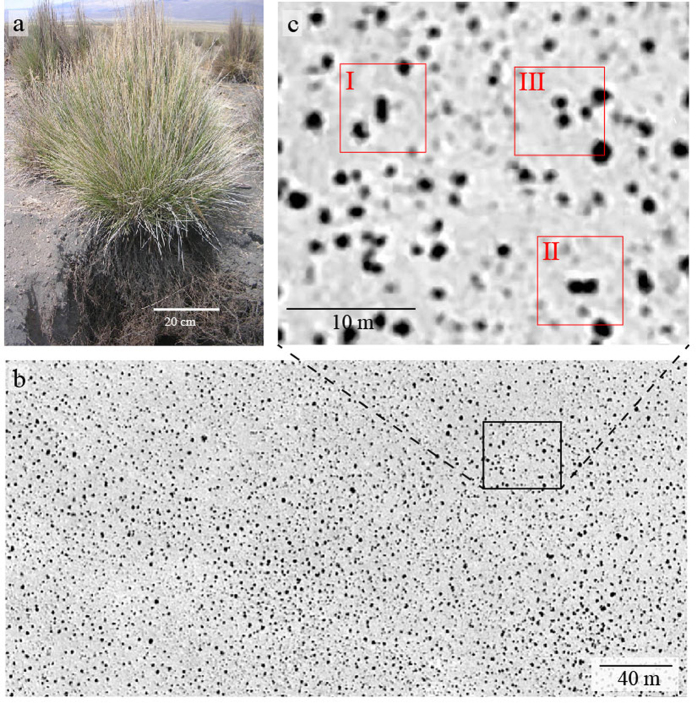
Study site in the Catamarca region, dark spots correspond to *Festuca orthophylla* tussocks. (**a**) Typical size tussocks of Festuca orthohylla in the Sajama National Park in the Bolivian Altiplano (courtesy of J.A. Fernandez Monteiro, Federal University of Sao Joao del Rei). (**b**) Processed image of the region under study, and (**c**) shows circular, elongated and splitting stages of a vegetation patch.

Festuca structures can be easily detected through satellite image analysis for their high light absorption (they appear as dark spots). As mentioned previously Festuca organises in tightly packed structures of circular shape. An example of isolated circular patch is shown in Fig. 2a. However, we have observed that an important number of structure have lost their circular shape, this curvature instability is the mechanism by which a tussock loses its circular shape by growing into an elliptical shape, the death of the tillers in the central part of the elongated structure causes the structure to split into two independent tussocks, we term this process self-replication, different stages of this process are shown by I, II, and III in Fig. 2c. This process is common to a wide range of species and scales, as observed in Fig. 1, where self-replications can be observed for structures in the scale of meters to hundreds of meters.

To address the question on how are Festuca tussocks distributed spatially, we perform a series of studies involving measurements of tussock properties and spatial distribution properties.

### Spatial distribution analysis. 

For studying the properties of the Festuca tussock, we have considered satellite imagery obtained directly from Google Earth Pro. For detecting Festuca patches, we performed image enhancement, which consisted on transforming the image to gray-scale, for the removal of background and improving contrast, a median filter and an adjustment of intensity was applied (Matlab R2105b), the resulting image can be seen in Fig. 2b. By turning the image to binary we could clearly identify the Tussocks. For the analysis objects in contact with the borders of the image were removed as they may not be completely observed, thus, introducing erroneous measurements to the analysis. The spatial resolution of the satellite images is 0.3 m, structures smaller than this size in diameter are not considered, however, this does not affect our spatial distribution analysis as we hypothesise that the spatial distribution of the vegetation will be dominated by the older/bigger tussocks as a consequence of their fully developed shallow root systems.

After the detection of the patches, the boundaries of each object and their properties (area, position, and radius) can be precisely computed. The relation of meters per pixel is extracted directly from the image (0.08 m/pixel). A total of 3204 structures where detected. The first analysis, corresponds to a detailed characterisation of small distance properties such as nearest neighbour distance (NND), area covered, and equivalent radius of each structure. The equivalent radius is obtaining by comparing the area of each structure with that of a circle of the same area. The nearest neighbour distance is computed as the minimal boundary-to-boundary distance, this will allow us to extract information on the relation between NND and root sphere size, which are useful in understanding the underlying pattern emerging from redistribution and competition for resources. To expose the emergent spatial order in tussocks distribution, spatial Fourier analysis was performed, its circular average allowed us to determine a characteristic wavenumber in the spatial system. For the effect of Fourier analysis, a square sub-figure was selected from the original one to avoid border size effects. Finally, we have computed the Voronoi tessellation for the centres of the structures. This analysis, suitable for studying the regularity of a pattern has been used previously in aerial analysis to address pattern formation in vegetation structures^61^. For both NND and Voronoi cell computation, a subset of structures where selected such that they where far enough from the images border to avoid error induced from non visible structures.

**Figure 3:**
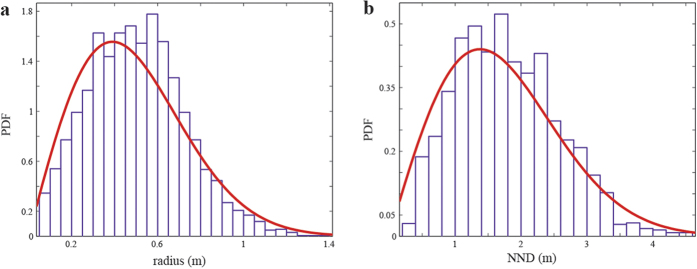
Probability density functions (PDFs) for the (**a**) equivalent radius of the structures and (**b**) the nearest neighbour distance (between edges). Red curves show Rayleigh distribution fits.

As stated previously, the remote sensing image analysis of the Catamarca region in NW-Argentina a total area of 109,44 km^2^ (384 m × 285 m) was studied. Structures found in the analysis ranged from an area of 0.09 m^2^ (minimum considered) to maximum area of 6.25 m^2^ with a mean of 0.95 m^2^ with s.d. of 0.79 m^2^. By visual inspection, we have noticed that bigger structures are most probably evolving clusters of structures. The average equivalent radius was found to be 0.50 m with s.d. of 0.22 m as can be seen in Fig. 3a. For calculating the minimal distance between the objects boundaries, only structures which are far enough from the image’s border are considered, as the objects not captured in the image could be closer to these structures than the other observable ones. By this consideration, distances between 2837 objects are viable, which vary from 0.25 m to 4.63 m, and averaged a distance of 1.83 m with s.d. of 0.77 m (see Fig. 3b).

Considering the average size and NND, and assuming that the distance between tussock is set under the constrain that the root spheres do not overlap, we can estimate the projected root sphere size of an average tussock as the half of the NND, this results in a root sphere of 1.4 m radius and 6.3 m^2^ area, which corresponds to an area of 6.7-fold the area of the average structure. This ratio is in fair agreement with the reported 6-fold ratio reported previously^60^ for *Festuca* in the Bolivian highlands, the 12% difference may suggest that vegetation is not efficiently covering the terrain, leaving fertile terrain unpolulated, this could be also an effect of the death cycle of tussocks.

Although the previous analysis give us insight on the properties of the structures and their distribution, it delivers no information on the spatial organization or the existence of a characteristic wavelength in the system, for this, we perform a Fourier analysis.

For the spatial Fourier analysis, we considered a square sub-figure of the original. This figure contained 1510 structures. The spatial Fourier transform is extensively used by pattern formation community to evaluate the degree of spatial organisation. Pronounced peaks in the 2D Fourier amplitude indicate not only the existence of characteristic lengths in the system but also a preferred spatial direction for the formation of the pattern.

**Figure 4:**
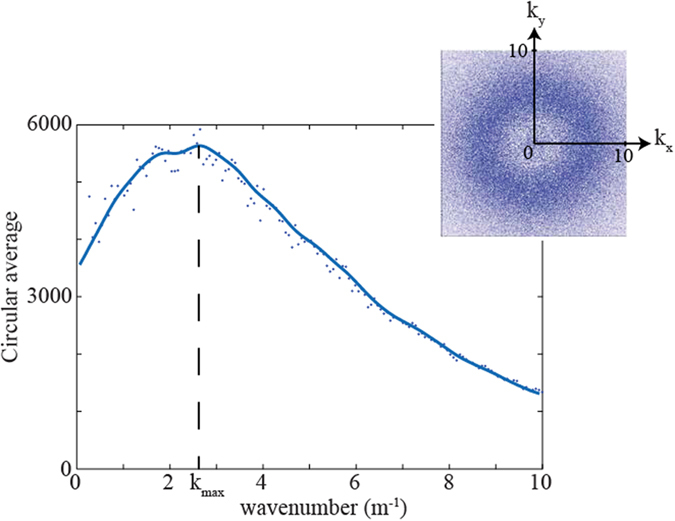
Spatial Fourier transform of the analysed data and its corresponding normalised circular average.

From the spatial Fourier analysis we are unable to detect pronounced peak in the spectrum, indicating that there is no preferred direction for a pattern to form. However, in the circular average of the spectrum, we observe a maximum wavenumber at k_*max*_ = 2.4 m^−1^ (see Fig. 4), this is the first sign that the system is arranging in such a way that a characteristic length L_*c*_ = 2*π*/k_*max*_ = 2.6 m emerges. As one would expect this characteristic length is related to the interaction between tussocks, no-overlap (between tussock root spheres) is achieved, in average, if the distances between the centres of the structures is at least 2.4 m according to estimations made in the previous section from the PDFs analysis. Root competition between plants generates a minimal distance between tussocks.

If we assume that the real ecosystem is self-organizing towards a state with a well defined characteristic length, then we should be able to observe some fingerprints of such a process in the macroscale.

**Figure 5:**
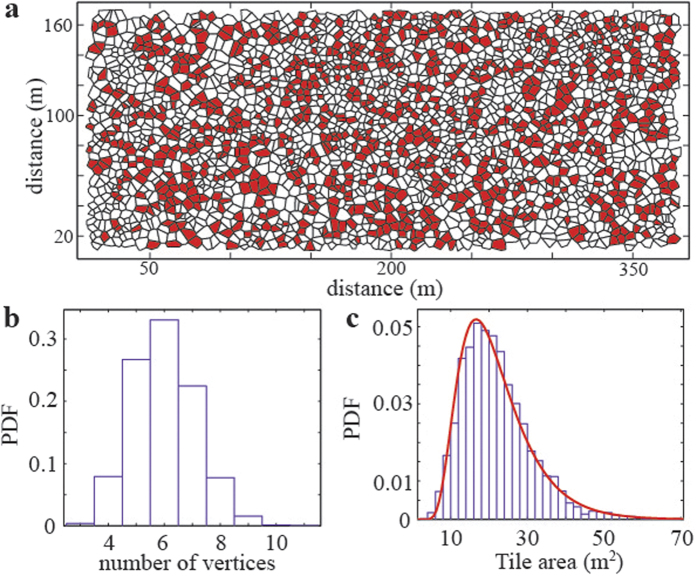
Field spatial information. From the analysed data we extract (**a**) Voronoi cell tessellation, 6-sided tiles are shown in red. (**b**) PDFs for number of vertices in each cell, and (**c**) tile area distribution with inverse Gaussian fit.

One of the problems of detecting such patterns and fingerprints in natural ecosystems is the existence of high scale perturbations such as terrain inhomogeneities, weather conditions, wind, and animal presence among others. All this external noise, alters the interactions between tussocks therefore, mangles their ability to form a regular pattern. The task of finding some type of unitary cell in the tussoks arrangement can be facilitated by the introduction of the Voronoi tessellation^62^. Considering the centre of a structure, the Voronoi cell associated with that structure will correspond to all the points that are closer to its centre than to the center of any other structure (see Fig. 5a). In this sense, the Voronoi tessellation gives us information of the most probable cell arrangement. As it is observed in Fig. 5b, the most probable vertex number is 6, which evidences an underlying tendency of Festuca tussocks to arrange spatially in hexagons. This is reinforced by observing that 6-sided cells seem not to be randomly distributed, rather forming cluster as if the hexagonal pattern was propagating through the system. Important information can also be extracted from the tile area (c.f. Fig. 5c), each tile represent the amount of land that is closer to a certain tussock than to any other, thus, the nutrients present in that portion of soil will be more accessible for the corresponding center tussock. The average tile size is 21.8 m^2^, which corresponds to equivalent radius of 2.63 m, almost twice the radius estimated for the root sphere. We know that when considering multiple structures, the roots spheres determine the minimum distance between them, however, the tile are observed indicates that tussocks are disperse through the terrain and still have space available for increasing the population density. We should remarked that the images considered do not give information of young and smaller tussocks that could be germinating on the suitable terrain.

In the next part of the article we present a simple model for describing pattern formation in the context of vegetation dynamics, the aim will be to theoretically describe the self-phenomena, and to show how important these phenomena is, both in obtaining a qualitative agreements between real field and numerical observations and as a mechanism for repopulating landscapes.

## Model for vegetation dynamics

Pattern formation in vegetated environments has been extensively studied both experimentally and theoretically. It is well known that competition for resources, such as, water and nutrients can lead to spatial self-organization. This behaviour favours the formation of a wide range of patterns that depend on the characteristics of both the environment and underground spatial distribution of roots.

Several models describing vegetation patterns and self-organization in arid and semiarid landscapes have been proposed during last two decades. They can be classified into three types. The first approach, often called generic interaction-redistribution models, are based on the relationship between the structure of individual plants and the facilitation-competition interactions existing within plant communities^4^,^5^,^6^,^7^,^8^,^9^,^10^,^11^,^12^,^13^. The second approach is based on the reaction-diffusion type of models. These take into account the influence of water transport by below ground diffusion and/or above ground run-off^14^,^15^,^16^,^17^,^18^,^19^,^20^,^21^,^22^. The third approach focuses on the role of environmental randomness as a source of noise-induced symmetry breaking transitions^23^,^24^,^26^.

In particular, the formation of localised structures in vegetation, also called localised vegetation patches has been studied in the case of poor resources, isotropic and homogeneous environments. A particular approach is to consider a logistic equation with a non-local term for describing the spatiotemporal evolution of the normalized biomass *b*(*r*, *t*) (the normalization is made with respect to the total amount of biomass supported by the system considered), this equation reads^31^


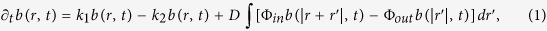


where *r* and *t* are the spatial coordinates and time, respectively. The factors *k*_1_ and *k*_2_ account for facilitation and competition mechanisms of the plant-to-plant feedbacks, respectively. The third term corresponds to seed dispersion mechanisms, *D* is the rate at which plants diffuse, and the kernels Φ_*in*_ and Φ_*out*_ weight the incoming and outgoing seed fluxes. The plant-to-plant interactions are considered to be of the form


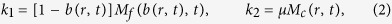


where


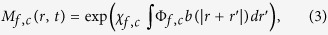


and the interaction strengths *ϕ*_*f*,*c*_ can be affected by both intrinsic and extrinsic factors^31^. The spatial extension of the plant-to-plant feedbacks are given by the kernels *ϕ*_*f*,*c*_, which in the case of the facilitation depend on the overground canopy which can provide a shelter for other plants to grow, conversely, the competition kernel depends on the root sphere size which depletes ground resources, preventing other vegetation to grow. In real vegetation the age, canopy size, and root sphere are related, as older and bigger plants require for a higher amount of nutrients, thus, increasing the root growth, this dependence of the root size on the above ground plant size is known as the allometric factor.

If we consider that the interaction kernels correspond to Gaussian fields, which do not depend on allometric factors, via a weak gradient approximation, equation (1) can be reduced to a local, non-variational partial differential equation for the phytomass density *ρ*(*r*, *t*)^27^, which reads


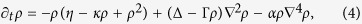


This equation contains three positive defined control parameters: *η* that account for the decrease-to-growth rate ratio; *κ* is the facilitation-to-competition susceptibility ratio; Δ is proportional to the square root of the facilitation-to-competition range ratio. The parameters Γ and *α* are the nonlinear diffusion coefficients. The real order-parameter equation (4) constitutes the simplest model of spatial dynamics in which competitive interactions between individuals occur locally. An important feature of this equation is the presence of nonlinear diffusion terms 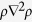 and 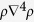, that render it non-gradient or nonvariational. These nonvariational terms are imputable to the dispersion process, if the dispersion is negligible then equation (4) is similar to the variational Swift-Hohenberg that is regularly derived in spatially extended systems. In that case, the coefficients of 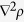 and 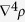 are both independent of the biomass density.

**Figure 6:**
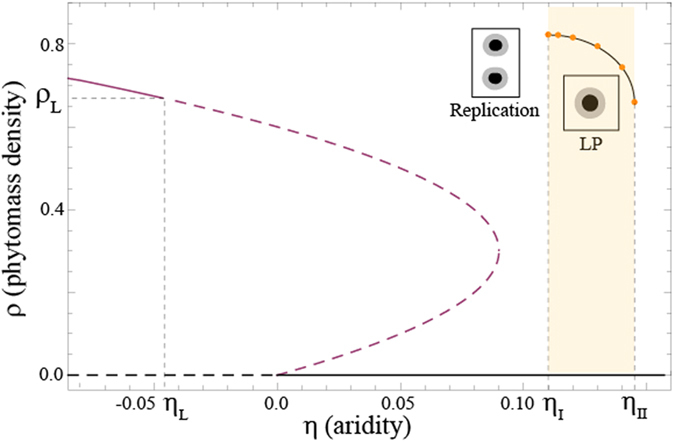
Bifurcation diagram of Eq. (4). Homogeneous vegetated states (purple and black), dashed lines indicate unstable regime. The stability curve for localized patches (LP) was constructed by direct simulation of Eq. (4) (orange dots). When aridity is decreased below *η*_*I*_ localized patches self-replicate.

The homogeneous steady-state solutions of Eq. (4) *ρ*_*s*_ are: (i) no plant state, 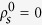, which corresponds to a territory devoid of vegetation, and (ii) an homogeneous plant population 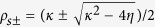 where at each point of the territory, the vegetation production and death are exactly balanced. They should be real and positive. Two situations must be distinguished according to the sign of *κ*. When *κ* ≤ 0, only the homogeneous steady state *ρ*_*s*+_, defines the biomass density, for *η* < 0. It decreases monotonously with *μ* and vanishes at *η* = 0. When *κ* > 0, the physical part of homogeneous branch of solution extends up to the limit point *ρ*_*L*_ = *κ*/2 and *η*_*L*_ = *κ*^2^/4. In the range 0 < *η* < *η*_*L*_, the biomass density exhibits a bistable behaviour: the stable homogeneous branches of solutions *ρ*_*s*−_ and *ρ*_*s*+_ coexist with the intermediate unstable branch 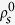 as shown in Fig. 6.

The upper homogeneous state 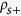 undergoes a modulational or spatial instability (Turing instability) characterised by an intrinsic wavelength


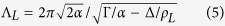


which measure the distance between two maxima or minima of the plant distribution. The threshold associated with the modulational instability is solution of the following cubic equation


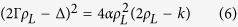


There exist more than one threshold associated with the modulational instability. In the following, we focus on parameter regime where the uniform plant distribution exhibit bistability (*κ* > 0) and a portion of this state becomes unstable with respect to the Turing bifurcation (for *η* > *η*_*L*_) as shown in Fig. 6. In this parameter range, any small fluctuation around the uniform plant distribution *ρ*_*s*+_ will trigger spontaneously the evolution of the system towards a stationary, spatially periodic distribution of the biomass density which will invade the whole territory. A detailed nonlinear analysis of two-dimensional periodic vegetation patterns such as stripes (often called tiger bush), and hexagons consisting of either sparsely populated or bare areas alternate with dense vegetations patches have been previously reported^5^.

It should be emphasised that the model presented is an approximation in which allometic factors are not considered, thus, the relation between the age and size of the plants with their interaction kernel is not considered. Moreover, the life-death cycle of the plants is not included in the theoretical construction of the model, this implies that a stable vegetation structure will persist in time without loss of phytomass.

Now we will show the mechanism by which a localized patch can lose stability to generate multiple localized patches.

## Self-replication as an extended pattern forming mechanism

**Figure 7:**
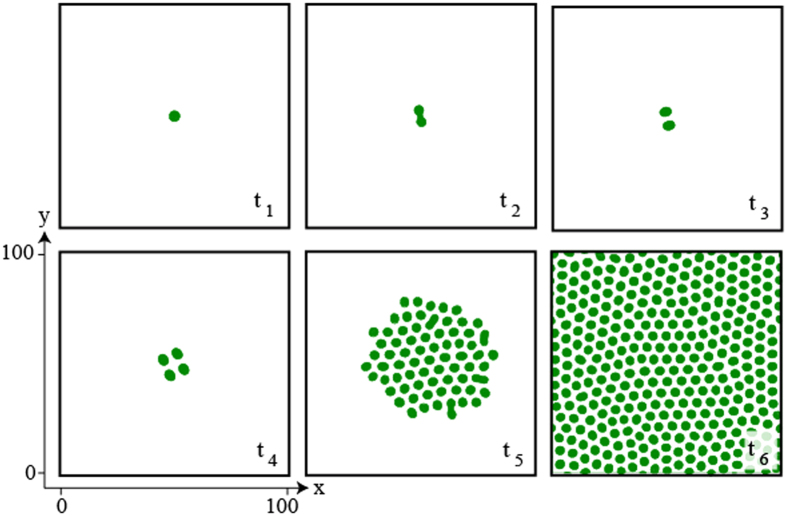
Localized patch self-replication. Temporal evolution of a localized patch of the vegetation model Eq. (4) for *η* = 0.1, *κ* = 0.6, Δ = 0.02 Γ = 0.5, and *α* = 0.125, integration grid 200 × 200, and periodic boundary conditions. Temporal evolution is from left to right panels, and from top to bottom ones, 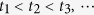. The localized patch suffers a curvature instability subsequently accompanied by the emergence of two spots. In turn these spots suffer a similar instability thus generating more spots, which begin to invade the system generating the emergence of a hexagonal pattern with several defects.

When increasing the aridity parameter *η*, i.e. decreasing the amount of resources available, the structures that appear first are gaps. They consist of spots of spare vegetation. They exist until they lose their stability towards the formation of localized vegetated patches. The region where these localised patches are stable is limited by aridity values *η*_*I*_ and *η*_*II*_ shown in Fig. 6. When a localised vegetation patch is stressed by decreasing the aridity below *η*_*I*_, the patch exhibits an elliptically deformation followed by its splitting as shown in Fig. 7.

This self-replicating process continues until the system is entirely occupied by spots. Only spots which have available space around them are able to replicate. Because of this, only spots located in the edges can replicate. In the real ecosystem available space can be generated by the death of a plant by natural or external perturbations (animals, fires), this is the reason we can observe self-replication throughout all the territory analysed previously.

For long time evolution, transition from a single patch to a self-organized hexagonal pattern through a self-replication phenomenon is shown in Fig. 7, defects in the biomass distribution are attributed to boundary conditions used to numerically simulate Eq. (4). This hexagonal regularity is not observed in the arrangement of Festuca tussocks observed in Fig. 2b as vegetation in a real ecosystem is not nucleated by a single spot but rather developed from the random seed spreading by wind and animals thus generating multiple tussocks in different locations, each with the possibility of splitting to spread through the terrain.

**Figure 8:**
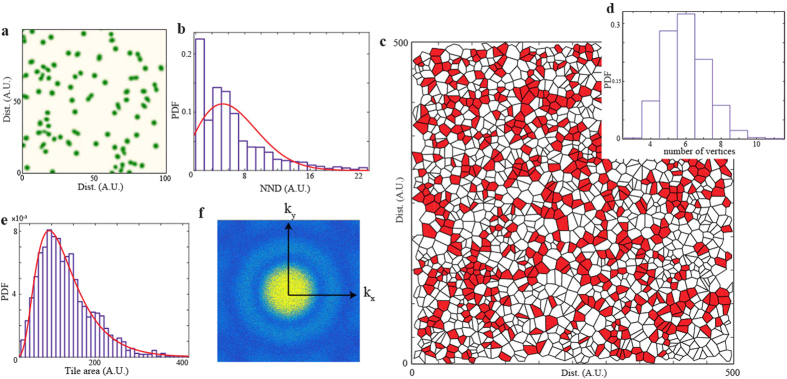
Extended field simulation. Numerical simulations of the non-variational phytomass model (4) for 1890 randomly distributed localized patches as initial conditions, each patch is in a different state of the self-replication process, with *η* = 0.1, *κ* = 0.6, Δ = 0.02 Γ = 0.5, and *α* = 0.125, integration grid of 1000 × 1000 with spacing *dx* = 0.5, and periodic boundary conditions. (**a**) Shows a portion of the system after a determined temporal evolution. Green areas represent vegetation patches. (**b**) PDF of the NND between the structures, with Rayleigh distribution fit. (**c**) Voronoi tessellation of the full system. 6-sided tiles are color red. (**d**,**e**) show the corresponding PDFs for the number of vertices in each cell, and tile area (with inverse Gaussian fit), respectively. (**f**) Fourier spectrum of the resulting field.

To study the importance of self replication in the large scale organisation and distribution of the phytomass, we consider the following numerical approach: we construct an initial condition given by a 1000 × 1000 points field, which contains 1849 randomly distributed vegetation patches, constructed by a two-dimensional Poisson point process with rate *r* = 0.002. This random distribution aims to mimic the natural long-range seed spreading mechanisms, such as, wind, birds, or terrestrial animals which can transport seeds through long distances, these factors are not considered in the local interaction-redistribution model, but could be incorporated in the general non-local logistic equation (1). For including life-cycle factors in our approach, we consider that 185 randomly selected structures are in some stage of the self-replication process, these range from single to fully split patches. To conserve isotropy and homogeneity the direction of the splitting of each patch is also chosen randomly. This artificially constucted field is then considered as the initial condition for the simulation of Eq. (4). The aridity level in the simulation is set bellow the *η*_*I*_ threshold, allowing each of the spots to continue the self-replication process, a portion of the resulting simulated field after 5000 iterations (with temporal step *dt* = 0.03) is shown in Fig. 8a. If we let the system evolve a sufficiently long time, then the system would reach an hexagonal pattern as observed in Fig. 7t6. However, when analysing the system in a intermediate state of evolution, we observe that the spatial distribution of the structures approaches qualitatively to the field observations of the Festuca tussocks in the Catamarca region, Argentina.

By computing the NND between the structures edges we obtain a PDF similar to that observed in the remote sensing analysis (see Fig. 8b). The dispersion around an average value of 9.5 A.U. (with a s.d. of 4.7) is generated by the self-replication process which alters the shape, consequently, the distance between structures. Structures at very small distances are not observed as the competitive interaction between spots causes a repelling force that generate a fast distancing between them once the splitting has occurred.

The Voronoi tessellation of the intermediate field shown in Fig. 8c exhibits a distribution where clusters of 6-sided cells are forming, in a system evolving towards and hexagonal organisation. In this stage, the tile vertex count (see Fig. 8d) and the cell area distributions (Fig. 8e) show that the characteristics previously observed in the field analysis are qualitatively well described by the generic interaction-redistribution model considered, under the initial conditions given.

The existence of the self-replication mechanism is fundamental in the spatial organization of the vegetation. If only a Poisson point process is considered for the location of the localized patches, without self-replication, similar distributions can be obtained, even for the Voronoi tessellations, however the Fourier transform of such field is homogeneous, showing no peaks, on the contrary, when giving the liberty of self-replication to the localized patches, immediately we observe the emergence of spatial organization, given by peaks in the Fourier transform, these peaks appear distributed in the form of a ring similar to the one exhibited by the field observations, however they differ in that the numerical one shows a wide central peak (see Fig. 8f).

Despite the simplicity of the model considered for the description of the vegetation dynamics, we have shown that the self-replication induced by the diminution of the aridity parameter and the distribution induced by competitive interactions, mediate the spatial organisation of the vegetation in semi-arid ecosystems.

## Conclusions

We have studied the self-replication phenomenon in the context of vegetation dynamics. Through remote sensing analysis of the Andean highlands we showed the existence of self-replication in Festuca tussocks, in this process the shrub is affected by a modulational instability that deform the structure from its circular shape into an elliptical shape, process after which the tussocks split into two new structures, we have also observed this process in a variety of species and size scales. By statistical analysis we have encounter characteristic distributions which are signatures of an underlying self-organization process. Though a general interaction-redistribution model that exhibits self-replication of localised structures we have shown that under certain initial conditions, the self replication and competitive interactions are sufficient conditions to exhibit spatial properties as the ones observed in natural ecosystems. These properties of the vegetation dynamical systems are the underlying mechanisms which mediate the extended self-organization of tussocks in arid and semi-arid ecosystems.

Self-replication in vegetation gives new lights on the way plants propagate and populate scarce environments.

## Additional Information

**How to cite this article**: Bordeu, I. *et al*. Self-replication of Localized Vegetation Patches in Scarce Environments. *Sci. Rep.* **6**, 33703; doi: 10.1038/srep33703 (2016).

## References

[b1] Greig-Smith P. Pattern in vegetation. The Journal of Ecology 755–779 (1979).

[b2] Levin S. A. The problem of pattern and scale in ecology: the Robert H. MacArthur award lecture. Ecology 73, 1943–1967 (1992).

[b3] Meron E. Nonlinear Physics of Ecosystems (CRC Press, Taylor & Francis Group, Boca Raton, 2015).

[b4] Lefever R. & Lejeune O. On the origin of tiger bush. Bulletin of Mathematical Biology 59, 263–294 (1997).

[b5] Lejeune O. & Tlidi M. A model for the explanation of vegetation stripes (tiger bush). Journal of Vegetation science 10, 201–208 (1999).

[b6] Gilad E., Von Hardenberg J., Provenzale A., Shachak M. & Meron E. Ecosystem engineers: from pattern formation to habitat creation. Physical Review Letters 93, 098105 (2004). 15447146 10.1103/PhysRevLett.93.098105

[b7] Barbier N., Couteron P., Lefever R., Deblauwe V. & Lejeune O. Spatial decoupling of facilitation and competition at the origin of gapped vegetation patterns. Ecology 89, 1521–1531 (2008). 18589517 10.1890/07-0365.1

[b8] Lefever R., Barbier N., Couteron P. & Lejeune O. Deeply gapped vegetation patterns: on crown/root allometry, criticality and desertification. Journal of Theoretical Biology 261, 194–209 (2009). 19651145 10.1016/j.jtbi.2009.07.030

[b9] Couteron P., Anthelme F., Clerc M. G., Escaff D., Fernandez-Oto C. & Tlidi M. Plant clonal morphologies and spatial patterns as self-organized responses to resource-limited environments. Philosophical Transactions of the Royal Society A: Mathematical, Physical and Engineering Sciences 372, 20140102 (2014). 10.1098/rsta.2014.0102 25246689

[b10] Grube S. The fairy circles of Kaokoland (Northwest Namibia) - is the harvester termite Hodotermes mossambicus the prime causal factor in circle formation? Basic and Applied Ecology 3, 367–370 (2002).

[b11] van Rooyen M. W., Theron G. K., van Rooyen N., Jankowitz W. J. & Matthews W. S. Mysterious circles in the Namib Desert: review of hypotheses on their origin. Journal of Arid Environments 57, 467–485 (2004).

[b12] Lefever R. & Turner J. W. A quantitative theory of vegetation patterns based on plant structure and the non-local F-KPP equation. Comptes Rendus Mécanique 340, 818–828 (2012).

[b13] Martínez-García R., Calabrese J. M., Hernández-García E. & López C. Vegetation pattern formation in semiarid systems without facilitative mechanisms. Geophysical Research Letters 40, 6143–6147 (2013).

[b14] Klausmeier C. A. Regular and irregular patterns in semiarid vegetation. Science 284, 1826–1828 (1999). 10364553 10.1126/science.284.5421.1826

[b15] Von Hardenberg J., Meron E., Shachak M. & Zarmi Y. Diversity of vegetation patterns and desertification. Physical Review Letters 87, 198101 (2001). 11690457 10.1103/PhysRevLett.87.198101

[b16] HilleRisLambers R., Rietkerk M., van den Bosch F., Prins H. H. & de Kroon H. Vegetation pattern formation in semi-arid grazing systems. Ecology 82, 50–61 (2001).

[b17] Okayasu T. & Aizawa Y. Systematic analysis of periodic vegetation patterns. Progress of Theoretical Physics 106, 705–720 (2001).

[b18] Rietkerk M., Dekker S. C., de Ruiter P. C. & van de Koppel J. Self-organized patchiness and catastrophic shifts in ecosystems. Science 305, 1926–1929 (2004). 15448261 10.1126/science.1101867

[b19] Meron E., Gilad E., von Hardenberg J., Shachak M. & Zarmi Y. Vegetation patterns along a rainfall gradient. Chaos, Solitons & Fractals 19, 367–376 (2004).

[b20] Sherratt J. A. An analysis of vegetation stripe formation in semi-arid landscapes. Journal of mathematical biology 51, 183–197 (2005). 15868198 10.1007/s00285-005-0319-5

[b21] Wang W., Liu Q-X. & Jin Z. Spatiotemporal complexity of a ratio-dependent predator-prey system. Physical Review E 75, 051913 (2007). 10.1103/PhysRevE.75.051913 17677104

[b22] Kéfi S., Rietkerk M., van Baalen M. & Loreau M. Local facilitation, bistability and transitions in arid ecosystems. Theoretical Population Biology 71, 367–379 (2007). 17097700 10.1016/j.tpb.2006.09.003

[b23] D’Odorico P., Laio F. & Ridolfi L. Patterns as indicators of productivity enhancement by facilitation and competition in dryland vegetation. Journal of Geophysical Research: Biogeosciences 111, 2005–2012 (2006).

[b24] D’Odorico P., Laio F., Porporato A., Ridolfi L. & Barbier N. Noise-induced vegetation patterns in fire prone savannas. Journal of Geophysical Research: Biogeosciences 112, 2005–2012 (2007).

[b25] Borgogno F., D’Odorico P., Laio F. & Ridolfi L. Mathematical models of vegetation pattern formation in ecohydrology. Reviews of Geophysics 47, (2009).

[b26] Ridolfi L., D’Odorico P. & Laio F. Noise-induced phenomena in the environmental sciences. Cambridge University Press (2011).

[b27] Lejeune O., Tlidi M. & Couteron P. Localized vegetation patches: a self-organized response to resource scarcity. Physical Review E 66, 010901 (2002). 10.1103/PhysRevE.66.010901 12241334

[b28] Meron E., Yizhaq H. & Gilad E. Localized structures in dryland vegetation: forms and functions. Chaos: An Interdisciplinary Journal of Nonlinear Science 17, 037109 (2007). 10.1063/1.2767246 17903016

[b29] Sheffer E., Yizhaq H., Shachak M. & Meron E. Mechanisms of vegetation-ring formation in water-limited systems. Journal of theoretical biology 273, 138–146 (2011). 21187102 10.1016/j.jtbi.2010.12.028

[b30] Escaff D., Fernandez-Oto C., Clerc M. G. & Tlidi M. Localized vegetation patterns, fairy circles, and localized patches in arid landscapes. Physical Review E 91, 022924 (2015). 10.1103/PhysRevE.91.022924 25768586

[b31] Tlidi M., Lefever R. & Vladimirov A. On vegetation clustering, localized bare soil spots and fairy circles. Lect. Notes. Phys. 751–381 (2008).

[b32] Dunkerley D. L. Infiltration rates and soil moisture in a groved mulga community near Alice Springs, arid central Australia: evidence for complex internal rainwater redistribution in a runoff-runon landscape. Journal of Arid Environments 51, 199–219 (2002).

[b33] Tlidi M., Staliunas K., Panajotov K., Vladimiorv A. G. & Clerc M. G. Localized structures in dissipative media: From Optics to Plant Ecology. Phil. Trans. R. Soc. A 372, 20140101 (2014). 10.1098/rsta.2014.0101 PMC4186218 25246688

[b34] Dickstein A. J., Erramilli S., Goldstein R. E., Jackson D. P. & Langer S. A. Labyrinthine pattern formation in magnetic fluids. Science 261, 1012–1015 (1993). 17739618 10.1126/science.261.5124.1012

[b35] Ribiere P. & Oswald P. Nucleation and growth of cholesteric fingers under electric field. Journal de Physique 51, 1703–1720 (1990).

[b36] Oswald P., Baudry J. & Pirkl S. Static and dynamic properties of cholesteric fingers in electric field. Physics Reports 337, 67–96 (2000).

[b37] Pearson J. E. Complex patterns in a simple system. Science 261, 189–192 (1993). 17829274 10.1126/science.261.5118.189

[b38] Lee K. J., McCormick W. D., Pearson J. E. & Swinney H. L. Experimental observation of self-replicating spots in a reaction-diffusion system. Nature 369, 215–218 (1994).

[b39] Muñuzuri A. P., Pérez-Villar V. & Markus M. Splitting of autowaves in an active medium. Physical review letters 79, 1941 (1997).

[b40] Kaminaga A., Vanag V. K. & Epstein I. R. A reaction–diffusion memory device. Angewandte Chemie International Edition 45, 3087–3089 (2006). 10.1002/anie.200600400 16570336

[b41] Kaminaga A., Vanag V. K. & Epstein I. R. “Black spots” in a surfactant-rich Belousov-Zhabotinsky reaction dispersed in a water-in-oil microemulsion system. Journal of Chemical Physics 122, 174706 (2005). 15910059 10.1063/1.1888386

[b42] Kolokolnikov T. & Tlidi M. Spot deformation and replication in the two-dimensional belousov-zhabotinski reaction in a water-in-oil microemulsion. Physical review letters 98, 188303 (2007). 17501615 10.1103/PhysRevLett.98.188303

[b43] Davies P. W., Blanchedeau P., Dulos E. & De Kepper P. Dividing blobs, chemical flowers, and patterned islands in a reaction-diffusion system. The Journal of Physical Chemistry A 102, 8236–8244 (1998).

[b44] Muratov C. B. & Osipov V. V. General theory of instabilities for patterns with sharp interfaces in reaction-diffusion systems. Physical Review E 53, 3101 (1996); Muratov, C. B., Osipov, V. V. Scenarios of domain pattern formation in a reaction-diffusion system. *Physical Review E* **54**, 4860 (1996); Muratov, C. B. Theory of domain patterns in systems with long-range interactions of Coulomb type. *Physical Review E* **66**, 066108 (2002). 10.1103/physreve.53.3101 9964617

[b45] Monine M., Pismen L., Bär M. & Or-Guil M. Modeling triangular titration fronts in the O2+ H2 reaction on a catalytic Rh (111) surface. The Journal of chemical physics 117, 4473–4478 (2002).

[b46] Schaak A. & Imbihl R. Triangular-shaped reaction fronts in a catalytic surface reaction. Chemical physics letters 283, 386–390 (1998).

[b47] Hayase Y. & Ohta T. Sierpinski gasket in a reaction-diffusion system. Physical review letters 81, 1726 (1998).

[b48] Hayase Y. & Ohta T. Self-replicating pulses and Sierpinski gaskets in excitable media. Physical Review E 62, 5998 (2000). 10.1103/physreve.62.5998 11101927

[b49] Meron E., Gilad E., von Hardenberg J., Shachak M. & Zarmi Y. Vegetation patterns along a rainfall gradient. Chaos, Solitons & Fractals 19, 367–376 (2004).

[b50] Ren X. & Wei J. On the spectra of three-dimensional lamellar solutions of the diblock copolymer problem. SIAM journal on mathematical analysis 35, 1–32 (2003).

[b51] Nishiura Y. & Suzuki H. Higher dimensional SLEP equation and applications to morphological stability in polymer problems. SIAM journal on mathematical analysis 36, 916–966 (2005).

[b52] Sandnes B., Knudsen H. A., Måløy K. J. & Flekkøy E. G. Labyrinth patterns in confined granular-fluid systems. Physical review letters 99, 038001 (2007). 17678328 10.1103/PhysRevLett.99.038001

[b53] Sandnes B., Flekkøy E. G., Knudsen H. A., Måløy K. J. & See H. Patterns and flow in frictional fluid dynamics. Nature communications 2, 288 (2011). 10.1038/ncomms1289 PMC3104512 21505444

[b54] Tlidi M., Vladimirov A. G. & Mandel P. Curvature instability in passive diffractive resonators. Physical review letters 89, 233901 (2002). 12485009 10.1103/PhysRevLett.89.233901

[b55] Hagberg A., Yochelis A., Yizhaq H., Elphick C., Pismen L. & Meron E. Linear and nonlinear front instabilities in bistable systems. Physica D: Nonlinear Phenomena 217, 186–192 (2006).

[b56] Bordeu I., Clerc M. G., Lefever R. & Tlidi M. From localized spots to the formation of invaginated labyrinthine structures in a Swift-Hohenberg model. Commun. Nonlinear Sci. Numer. Simulat. 29, 482 (2015).

[b57] New M., Lister D., Hulme M. & Makin I. A high-resolution data set of surface climate over global land areas. Climate Research 21, 1–25 (2002).

[b58] Ospina González J. C., Aliscioni S. S. & Denham S. S. Estudios taxonómicos en el género Festuca L.(Poaceae) de Argentina y Chile. Gayana. Botánica 70, 01–15 (2013).

[b59] Monteiro J. A. F. Functional morphology and productivity of a Tussock grassland in the Bolivian Altiplano (*Doctoral dissertation, University of Basel*) (2012).

[b60] Monteiro J. A. F., Hiltbrunner E. & Körner C. Functional morphology and microclimate of Festuca orthophylla, the dominant tall tussock grass in the Andean Altiplano. Flora-Morphology, Distribution, Functional Ecology of Plants 206, 387–396 (2011).

[b61] Getzin S., Wiegand K., Wiegand T., Yizhaq H., von Hardenberg J. & Meron E. Adopting a spatially explicit perspective to study the mysterious fairy circles of Namibia. Ecography 38, 1–11 (2015).

[b62] Aurenhammer F. Voronoi diagrams—a survey of a fundamental geometric data structure. ACM Computing Surveys (CSUR) 23, 345–405 (1991).

